# Guy's Hospital Reports

**Published:** 1892-12

**Authors:** 


*Guy's Hospital Reports*. Edited by N. Davies-Colley, M.A., M.C., and W. Hale White, M.D. Volume XLVIII. Pp. xxxiii., 386. London: J. & A. Churchill. 1892.

Of the numerous original papers in this volume, that by Dr. Frederick Taylor "On Malignant Endocarditis" is most worthy of detailed examination, in consequence of the fact that the disease is not yet sufficiently understood and is frequently not recognised. The diagnosis from typhoid fever, pyæmia, ague, and general tuberculosis is not at first possible, and it has been pointed out that there is no organism peculiar to the disease. It appears that ulcerative and malignant endocarditis is liable to succeed every now and then to a preceding acute disease such as pneumonia, and that chronic endocarditis is liable to take an ulcerative form in hospitals where such diseases as pyæmia, typhoid, or scarlatina may be rife. The important clinical features—namely, the cardiac murmurs, the pyrexia, and the evidence of embolic processes—are described in detail. Prognosis does not appear to be absolutely hopeless, and possibly the bacteriologist of the future may provide some more adequate treatment than that of the symptomatic indications which are followed to little purpose. An analysis of 54 cases of the disease concludes this interesting and instructive essay.

The other papers maintain the usual standard of excellence which always characterises the work of Guy's Hospital.

